# Numerical studies on the seismic response of support hanger for building pipelines

**DOI:** 10.1371/journal.pone.0324077

**Published:** 2025-05-28

**Authors:** Min Huang, Jun He, Jun Liu, Junsheng Huang, Wenbin Pan, Hao Wang

**Affiliations:** 1 China Construction Science and Industry Corporation Ltd., Shenzhen, China; 2 School of Civil Engineering, Tianjin Chengjian University, Tianjin, China; 3 School of Civil Engineering, Tianjin University, Tianjin, China; Universita degli Studi di Napoli Federico II, ITALY

## Abstract

This study presents the development of numerical models that integrate wire, shell, solid, and connector elements to assess the mechanical performance of support hanger systems under both pushover and seismic loading conditions. Specifically, the models incorporate axial connectors with low compression stiffness and high-tension stiffness to simulate the tension-only behavior characteristic of tension bars. Additionally, the seismic analysis models account for the influence of pipes. Utilizing these models, parametric studies were conducted to investigate the impact of key parameters, including connector thickness, connector angle, brace angle, spacing of support hanger, earthquake waves, and the peak acceleration. The findings provide valuable insights for researchers and engineers in the field.

## Introduction

Earthquake disasters can result in significant casualties and economic losses, making seismic design a crucial aspect of building structure design [1]. However, current structural seismic design primarily focuses on the response of load-bearing structures and components to earthquakes [2], while non-load-bearing structures (or components) are often simplified as load forms and lack of specific seismic design and analysis [3–5]. In the case of small to moderate earthquakes, the destruction of non-structural components frequently accounts for a substantial portion of casualties and economic losses [6]. Although structures and load-bearing components designed for seismic resistance often remain intact during such events, avoiding catastrophic damage, the collapse of non-load-bearing structures or components that have not been seismically designed can lead to both direct economic losses and, tragically, casualties [7,8].

For safety design purposes, researchers have conducted experimental and theoretical studies on the mechanical performance of non-load bearing structures [9–11]. Badillo et al. [12] investigated the seismic fragility of suspended ceiling systems, highlighting that the use of compression members can enhance their seismic performance and proposed fragility curves for four damage states. Ryu et al. [13] reported on a series of full-scale dynamic tests of large area suspended ceiling systems, discovering that lateral restraints can improve performance. Tian et al. [14,15] performed dynamic tests and numerical simulations on full-scale support hanger for pressurized sprinkler piping, indicating that braced systems significantly outperform unbraced ones. They also developed a hysteretic model to account for changes in strength and stiffness during the unloading and reloading of full-scale sprinkler joints. Song et al. [16] conducted a quasi-static test on longitudinal support hanger, emphasizing that the design and quality of connectors are pivotal to seismic performance. Zheng et al. [17] conducted shaking table tests on support hanger, showing that it can markedly reduce the displacement of piping systems, with a vibration reduction ratio of up to 96%, although this effect was primarily observed under large accelerations. Li et al. [18] suggested using steel cables in place of braces to increase lateral stiffness and resistance, and they numerically simulated the mechanical performance of this new type of support hanger under hysteretic loading as specified by GB 50981 [19] and GB/T 37267 [20]. As describe above, the existing researches on support hanger mechanical performance primarily focuses on specific cases and lacks a comprehensive investigation into the influence of components and the interactions between pipes and structures, and the numerical models were based on solid elements or wire elements. Where the solid models contain too much details, a large calculation resource is necessary, while the wire models were too simplified by ignored some details, especially for the connector parts. This gap in the literature underscores the need for a more holistic approach to understanding and predicting the behavior of support hangers under various loading conditions.

To fill this gap, the authors have developed an innovative finite element model to predict the mechanical behavior of support hangers under both pushover and seismic loading conditions. In the developed model, a combination of solid elements, shell elements, wire elements, and connectors was employed to accurately represent the bracing system while maintaining a balance between computational efficiency and modeling precision. Based on the developed model, a comprehensive parametric study has been conducted to analyze the response of support hangers under pushover and seismic loading scenarios. With the results, the influence of the various parameters, connector thickness, connector angle, brace angle, design load, seismic wave characteristics, and peak acceleration on the pushover and seismic performance of bracing systems were investigated.

### Finite element model (FEM)

#### Brief of the numerical model.

**Fig 1:**
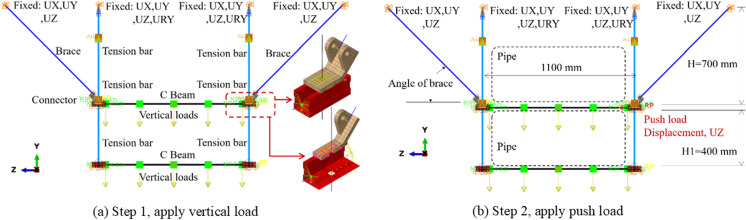
Details of numerical model for pushover analysis.

**Fig 2:**
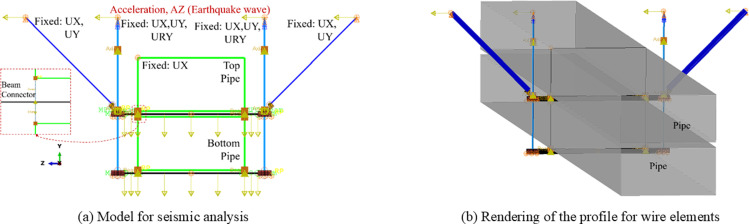
Details of numerical model for seismic analysis.

To evaluate the mechanical performance of support hanger for building pipelines, simulations of both pushover and seismic behaviors have been conducted, with the corresponding models depicted in Figs 1 and 2, respectively. The pushover behavior simulation is grounded in quasi-static analysis, wherein the pipes are assumed to move in tandem with the support hanger without exerting reaction forces. Consequently, it is deemed unnecessary to incorporate the pipes into the models for this analysis. In contrast, seismic behaviors are simulated through transient dynamic analysis, where acceleration induces inertial forces on the pipes. For components are included in the model, the mass of element will automatedly calculated by the volume of element and the corresponding density, while for the model without element for the pipe line, the equivalent non-structural mass is applied on the C beam, marked with squares in the see Fig 1a. It is therefore advisable to include the pipes in the numerical model to account for their influence, particularly for long pipes that span the space between two support hangers.

**Fig 3:**
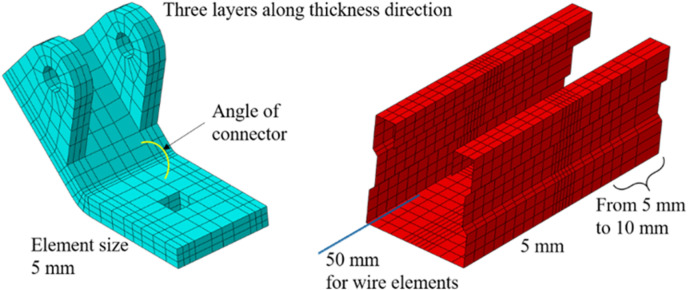
Mesh of the shell and solid parts.

As shown in Figs 1 and 2, the numerical model is executed in two distinct steps: the first step involves applying a vertical load, while the second is designed to apply lateral loadings. In the context of pushover analysis, this lateral loading is represented by a displacement at the load set point. For seismic analysis, the lateral loading is defined by the acceleration (time-history curve derived from the earthquake wave) applied to the four constraint points. It is evident that the tension bars, braces, and the central segments of C Beams (excluding the ends) are modeled using B32 elements (3-node quadratic beam in space). The ends of the C Beams are constructed with S4R shell elements (4-node doubly curved thin or thick shell, reduced integration, hourglass control, finite membrane strains), and the connectors are represented by C3D8R solid elements (8-node linear brick, reduced integration, hourglass control) to include more details. Furthermore, the maximum element size for the linear elements is specified as 50 mm. The typical mesh for the shell and solid components are presented in Fig 3.

#### Interactions and connector element.

**Fig 4:**
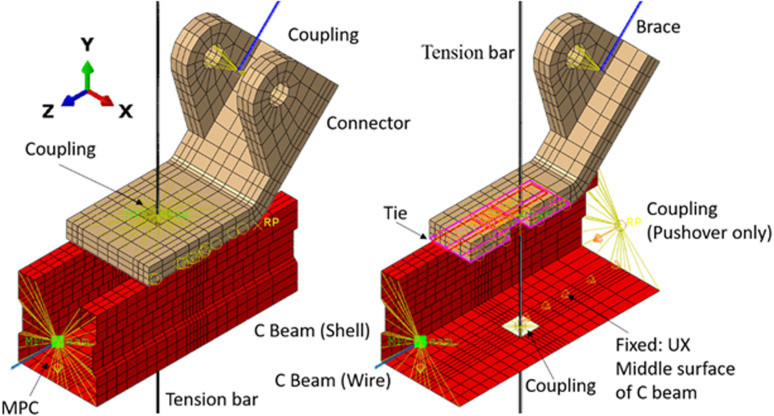
Interactions between components.

To simplify the numerical model, various interactions, including ties, couplings, and MPCs (multi-point constraints), are incorporated. These interactions, particularly around the connector, are detailed in Fig 4 and Table 1 where: 1) tie is a rig connection for two surfaces without slip; 2) the coupling or MPC is used to create a rig body for the main node and the slave nodes (nodes on the constraint surfaces or edges), and its behaviors are described by the main node; 3) BEAM connector and AXIAL connector are two types of connect elements, for BEAM connector, there is no relative deformation of the connected two nodes, while for AXIAL connector, displacement along the axis is allowed, and its linear or non-linear behavior can be described by a displacement-force curve.

**Table 1:** Details of the interactions in numerical models.

Master region	Slaver region	Interactions	Remark
Bottom surface of connector	Top surface of C beam	Tie	The contact zone
Vertice at the end vertice of tension bar	Top surface of the connector	Coupling	The flat surface only
Vertice at the end of C beam (wire)	Edges at the end section of the C beam (shell)	MPC	–
Reference point (for push load)	Edges at the end section of the C beam (shell)	Coupling	Only for pushover analysis
Vertice at the end of brace	Cylinder surfaces of the connector	Coupling	Exclude the degree of URX
The key point on tension bar	Edges on the C beam (shell) around the key point	Coupling	–
Vertice at the corner of pipe	Vertice at the C beam (wire)	BEAM connector	Only for seismic analysis

MPC is the abbreviation of multi-points constraints, and URX means the rotation freedom around the X axis.

For the tension bars, the compressive resistance is considered negligible due to their minimal bending stiffness. A tension-only constitutive model is suitable for simulating this behavior; however, it may result in convergence issues. Consequently, the authors propose the use of AXIAL connectors to model these bars, except for the tension bars beneath the top C beam, as shown in Figs 1 and 2. These AXIAL connectors are characterized by a very low compression stiffness and a high tension stiffness.

#### Constitutive law.

**Fig 5:**
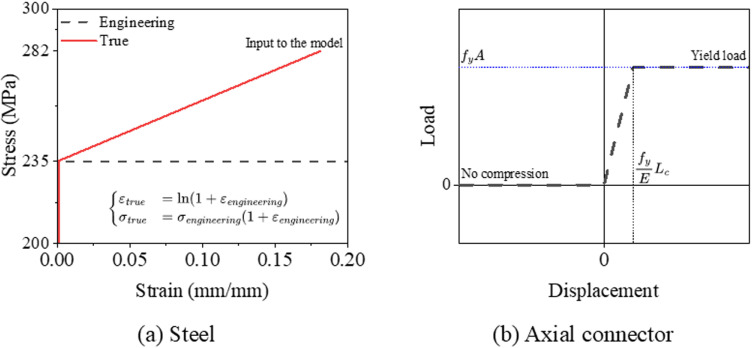
Constitutive models.

The elastic-plastic stress-strain model is applied to describe the mechanical behavior of steel components, such as bracings, C-beams, and connectors. According to the requirement of the software, the engineering stress-strain data should be converted to true stress-strain data before input to the model, as shown in Fig 5a. While for the axial connector elements, the displacement-load curve, as shown in Fig 5b, is defined to describe the no compression characteristic of tension bar. Where the $f_{y}$, E are the yield stress and Young’s model of the material, respectively; A and $L_{c}$ are the area and length of the connector, respectively.

#### Geometry imperfection.

**Fig 6:**
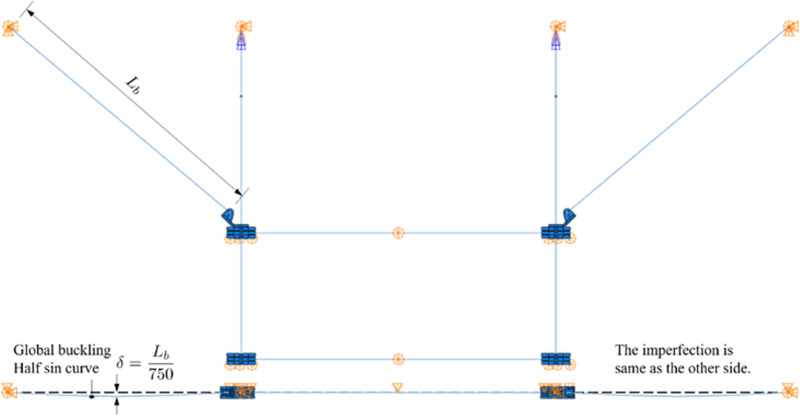
Illustration of the introduced geometry imperfection (*L*_*b*_ and *δ*  are the length and imperfection of the support hanger, respectively.

For both types of loading cases, some members will be suffered compression, which may lead to buckling. Due to the out-plane displacement of C Beams are constrained by the pipe line, the support hangers are more prone to buckling. To mitigate this issue, an imperfection modeled as a half-sine wave curve (global buckling model of the corresponding component) is incorporated, with a maximum geometric deviation of 1/750 of the support structure’s length [21], as shown in Fig 6.

### Pushover analysis

The geometric and material properties of the components for the control model are detailed in Table 2. It is important to note that an elastic-plastic stress-strain model is employed to simulate the mechanical behavior of the steel. In accordance with the Chinese standard for the design of steel structures [22], the Young’s modulus and Poisson’s ratio are specified as 206 GPa and 0.3, respectively. To investigate the influence of key parameters, such as connector dimensions (angle and thickness), brace angle, and the spacing of support hanger, a parametric study is conducted.

**Table 2:** Details for the control models.

Member	Dimension	Material	Weight (kg/m)
C-Beam	C41 × 41 × 2	Q235	2.423
Bracing	C41 × 41 × 2	Q235	2.423
Tension bar	D12	Q235	0.222
Connector	8 mm, 135^∘^	Q235	–
Pipe	B800 × 400 × 1	Q235	18.84 (≈188.4 N/m)

#### Connector angle.

**Fig 7:**
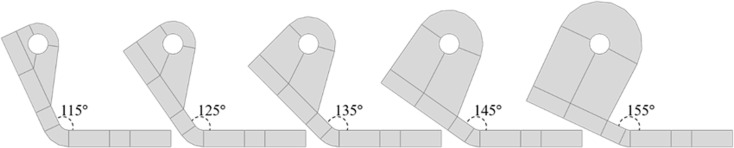
Connector with various angles.

Fig 7 illustrates connectors with varying angles, which are designed to connect with the support hanger in practical applications. To eliminate the influence of changes in the connection point, all connectors the same hole size and position.

**Fig 8:**
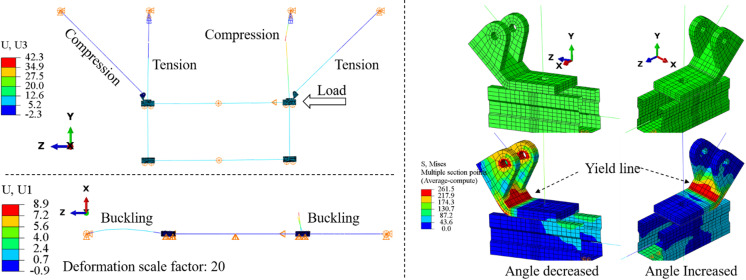
Contours of the typical support hanger (The control model).

Fig 8 the failure mode of the support hanger under a push load. A significant out-of-plane deformation (along the X direction) is observed, which is caused by the buckling of the compression brace and the compression tension bar. Additionally, a pronounced yield line is evident on the connector, attributed to the bending moment. For the left connector, the bending moment is induced by the compressive load from the brace, resulting in a decrease in the connector’s angle. Conversely, for the right connector, the bending moment originates from the tension load of the corresponding brace, leading to an increase in the connector’s angle.

**Fig 9:**
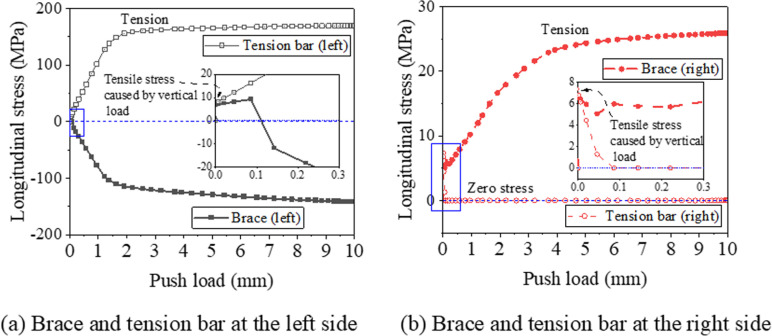
Longitudinal stress of braces and tension bars (The control model).

Fig 9 illustrates the longitudinal stress distributions in the braces and tension bars. As shown in Fig 9a, on the left side, the brace experiences compression while the tension bar is under tension; the stress values (with absolute values taken, where negative indicates compression) increase with the increase in push load (lateral displacement). Conversely, on the right side, the brace and tension bar are under tension and compression, respectively, as shown in Fig 9b. This stress distribution is the exact opposite of that on the left side and is significantly lower. For the brace, the tension stress is less than 30 MPa, and the longitudinal stress of the tension bar is zero when the horizontal displacement is equal to or greater than 0.1 mm. This observation indicates that the proposed method effectively captures the tension-only behavior of the tension bars. Furthermore, the initial tension stress in the four members is induced by the vertical load.

**Fig 10:**
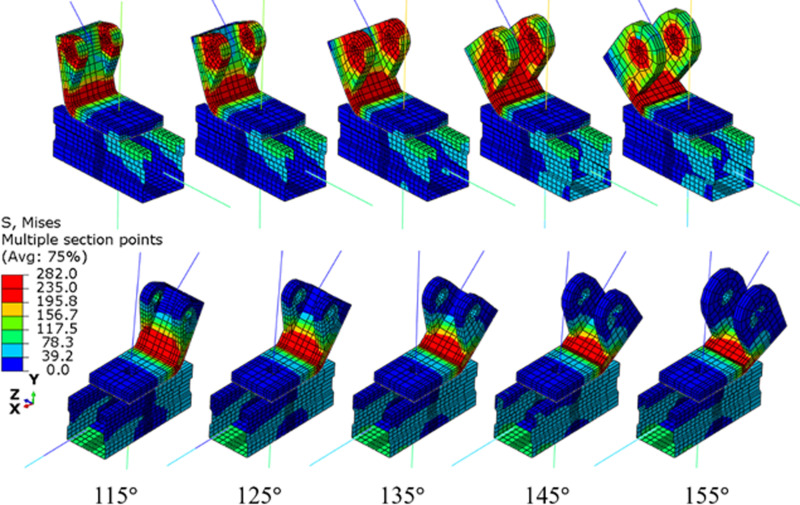
Von mises stress contours for models with different connector angles.

Fig 10 presents a comparison of the von Mises stress contours across different models. Consistent with the control model, distinct yield lines are evident at the corners of the connector. This observation suggests that augmenting the connector’s angle from 115° to 155° does not alter the failure mode of the support hanger.

**Fig 11:**
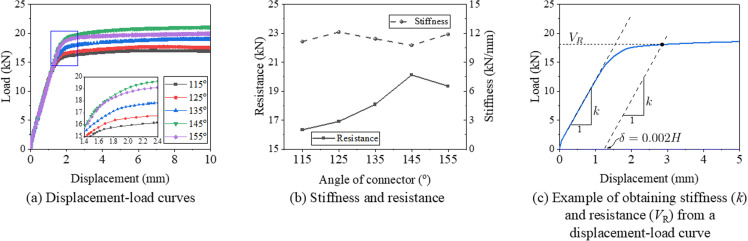
Displacement-load curve and resistance of support hanger with various connector angle.

Fig 11 compares the displacement-load curves, stiffness, and resistance for samples with various connector angles. On the one hand, due to the geometry imperfection, the initial stage of the displacement-load curve is not a linear, thus a linear stage after that is suggested to calibrate the stiffness. One other hand, considered the yield behavior of components, the maximum may overestimate its design resistance. Reference to the definition of the proof stress at a plastic strain of 0.2% of steel, the authors suggested that use the load at a plastic displacement of 0.2%H as the resistance of the structure, as illustrated in Fig 11c. Fig 11a reveals that all samples exhibit a similar initial stiffness, with the primary differences in the displacement-load curves occurring during the plateau stage. Fig 11b presents a comparison of stiffness and resistance values. It is evident that the stiffness for all samples ranges from 10.7 kN/mm at a 145° connector angle to 12.1 kN/mm at a 125° connector angle, with a difference of only 1.4 kN/mm between the maximum and minimum values. However, as the connector angle increases from 115° to 145°, the resistance increases from 16.3 kN to 20.1 kN, marking a 23.1% increase. In contrast, when the connector angle increases from 145° to 155°, the resistance decreases slightly to 19.9 kN, a reduction of 1.0%. Based on these findings from the control model components, a connector angle between 135° and 155° is recommended.

#### Connector thickness.

**Fig 12:**
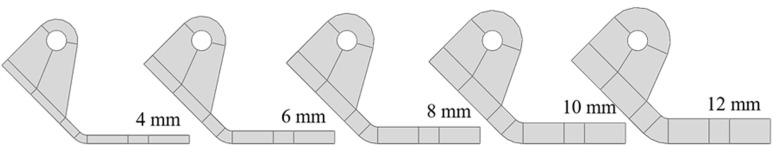
Connector with different thicknesses.

**Fig 13:**
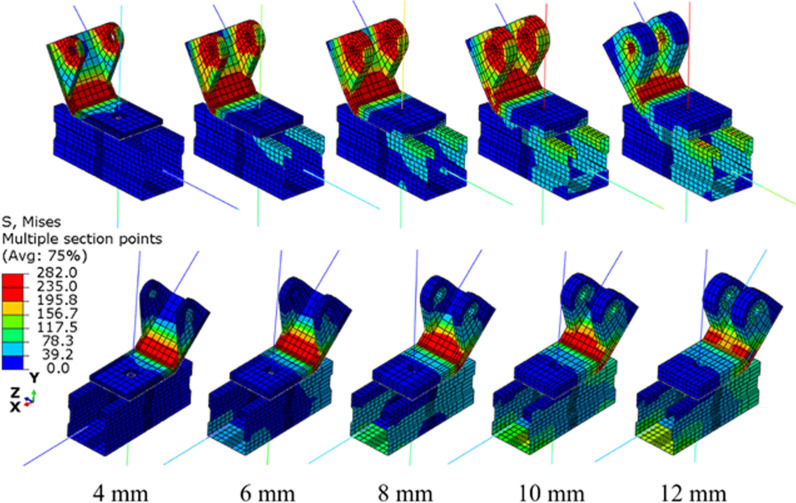
Von mises stress contours for models with different connector thicknesses.

Fig 12 shows connectors with varying thicknesses, ranging from 4 mm to 12 mm, all of which maintain the same circular hole size and position. Fig 13 presents a comparison of the von Mises stress contours for these connectors across different models. It is evident that distinct yield lines are observed at the corners of the connectors, except for the 12 mm case. This observation suggests that as the thickness increases, the dominant failure mode of the sample shifts from connector yielding to brace buckling.

**Fig 14:**
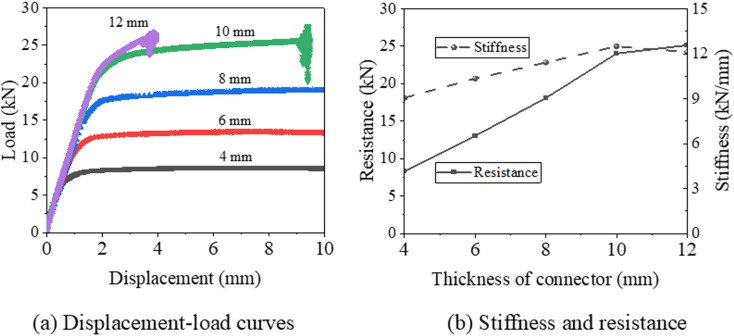
Displacement-load curve and resistance of support hanger with various connector thicknes.

Fig 14 compares the displacement-load curves, stiffness, and resistance of samples with various connector thicknesses. As shown in Fig 14a, for samples with a thickness greater than 8 mm, a sharp drop at the end of the displacement-load curve is observed, which is attributed to the buckling of the brace. In contrast, for samples with thinner connectors (thickness not exceeding 8 mm), the displacement-load curves exhibit a significant plateau stage following the yield point. Fig 14b presents the comparative analysis of stiffness and resistance. It is observed that as the connector thickness increases from 4 mm to 10 mm, the resistance and stiffness increase from (8.3 kN, 9.1 kN/mm) to (24.0 kN, 12.5 kN/mm), representing an increase of 37.9% and 189.5%, respectively. However, when the connector thickness increases from 10 mm to 12 mm, the resistance increases by only 1.1 kN, and the stiffness actually decreases by 0.4 kN/mm. Based on these findings from the control model components, a connector thickness ranging from 5 mm [20] to 10 mm is recommended.

#### Brace angle.

**Fig 15:**
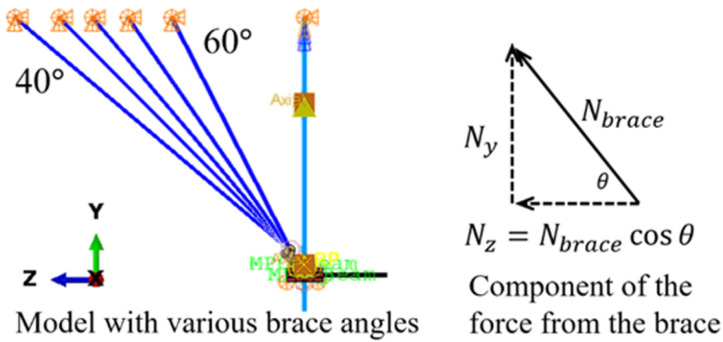
Models with different brace angles.

**Fig 16:**
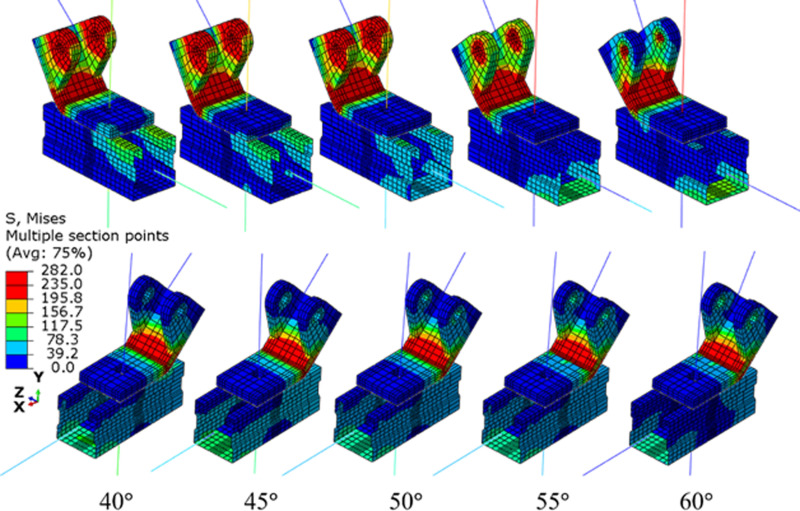
Von mises stress contours for models with different brace angles.

Fig 15 shows the model with brace angles (θ) ranging from 40^∘^ to 60^∘^. Fig 16 presents a comparison of the von Mises stress contours for these different models. It is evident that distinct yield lines are observed at the corners of the connectors. However, the yield area near the circular hole decreases as the brace angle increases. For the sample with a brace angle of 60°, yielding is confined primarily to the elements around the hole. This trend is attributed to the fact that with a larger θ, the cosine of θ and the force component ($N_{z}$) are smaller, as shown in Fig 16.

**Fig 17:**
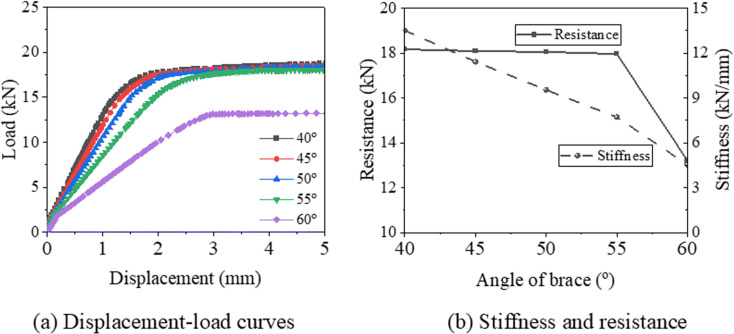
Displacement-load curve and resistance of support hanger with various brace angle.

Fig 17 compares the displacement-load curves, stiffness, and resistance of samples with various brace angles. As shown in Fig 17a, a larger brace angle correlates with lower stiffness, yet all samples exhibit a similar plateau stage, except for the sample with a brace angle of 60°. When the brace angle increases from 40° to 55°, the resistance of the support hanger decreases, but its horizontal component remains greater than the yield load of the connector. Consequently, the maximum load for these samples is very close. However, for the 60° case, the horizontal component is less than the yield load of the connector, and the failure mode is controlled by the buckling of the brace, resulting in a significantly lower maximum load compared to other samples. Fig 17b illustrates that as the brace angle increases from 40° to 60°, stiffness decreases from 13.5 kN/mm to 4.5 kN/mm, a reduction of 66.5%. Furthermore, while the increase in resistance from 40° to 55° is only -1.4 kN, the increase from 55° to 60° is -4.7 kN. Based on these findings from the control model components, a brace angle ranging from 40° to 55° is recommended.

#### Space of support hanger.

**Fig 18:**
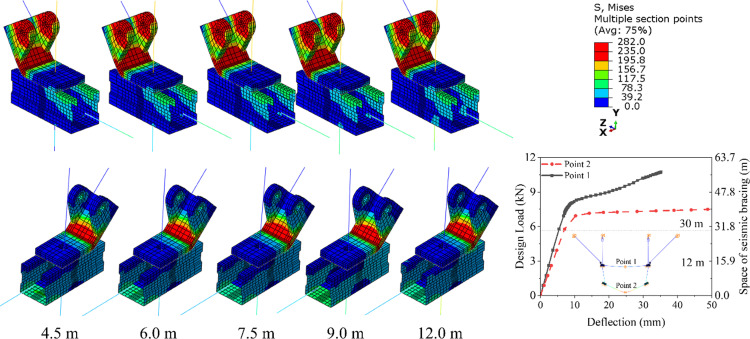
Von mises stress contours for models with different space of support hanger.

The design load for support hanger is primarily derived from the pipes. Consequently, the greater the spacing of support hanger—defined as the length of the pipe between two consecutive support hangers—the higher the design load will be. Fig 18 summarizes the von Mises stress contours of the connectors. It is observed that there is negligible difference in the location and area of the yield zone, as the stress induced by the design load is relatively small. As shown in Fig 9, for the control model, which has a support hanger spacing of 9 meters, the longitudinal stress in the braces and tension bars ranges from 6 MPa to 10 MPa, significantly lower than the yield stress of 235 MPa. Furthermore, Fig 18 also shows the comparison of the deflection-load curves of the middle points on the top and bottom C beams. It is easy to find that the bottom C beam (Point 2 in Fig 18) exhibits a greater deflection than the top C beam (Point 1 in Fig 18), and when the load exceeds 6 kN, the deflection of the bottom C beam increases fast with the increases of loading. It indicates that for the study case, the structure can resistant a vertical load of 6 kN, which means the pipe line with a length longer than 30 m.

**Fig 19:**
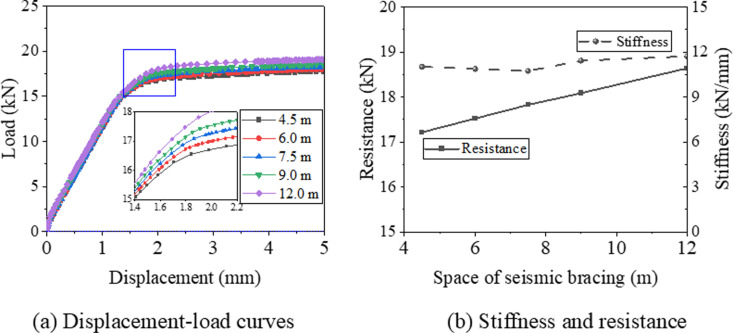
Displacement-load curve and resistance of support hanger subjected different design load.

Fig 19 summarizes the displacement-load curves, stiffness, and resistance of samples with varying pipe lengths. It is evident that both the maximum load and resistance increase as the spacing between support hangers expands. Specifically, as the spacing increases from 4.5 m to 12 m, the resistance rises from 17.2 kN to 18.6 kN, marking an increase of 8.1%. This enhancement in resistance is attributed to the increased tensile stress resulting from the design load.

### Seismic analysis

**Fig 20:**
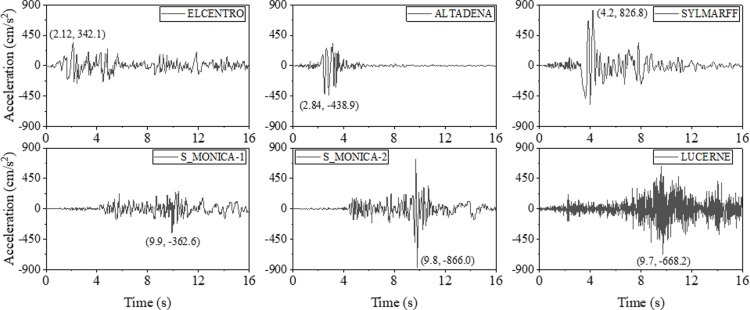
Typical earthquake waves.

**Fig 21:**
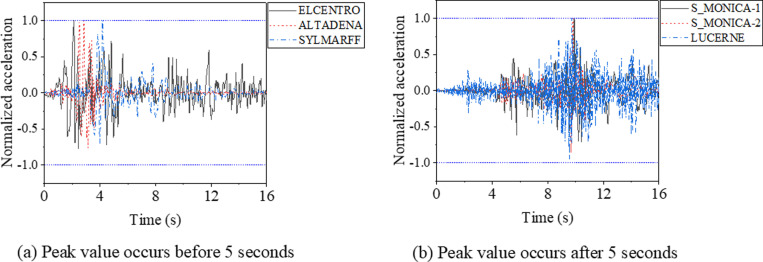
Normalized earthquake waves (with a maximum value of unit one).

To evaluate the seismic behavior of support hanger, six earthquake waves were collected, as shown in Fig 20. Analysis reveals that the peak accelerations of the ELCENTRO, ALTADENA, and SYLMARFF waves occur before 5 seconds, whereas for the S_MONICA-1, S_MONICA-2, and LUCERNE waves, the peak accelerations occur around the 10th second. Furthermore, to mitigate the influence of peak acceleration variations, normalized earthquake waves are employed in the analysis, as illustrated in Fig 21.

#### Earthquake waves.

According to the specifications outlined in GB 50011-2010 [1], for regions classified with a seismic fortification intensity ranging from six to nine degrees, the design basic earthquake acceleration should be between 0.05g and 0.40g, where g represents the acceleration due to gravity. Specifically, for areas designated with a seismic fortification intensity of seven degrees, the design basic earthquake acceleration is specified as either 0.10g or 0.15g, with the precise value being contingent upon the type of soil present. Consequently, this study selects 0.10g as the design basic earthquake acceleration for its analysis. It is worth to mention that all the model in this section are performance by non-linear time history analysis by using the explicit solver.

**Fig 22:**
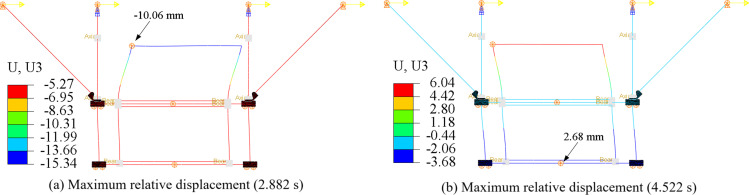
Displacement contours of the control model under ELCENTRO wave (Deformation scale factor: 10).

Fig 22 presents the displacement contours of the control model at the instance when the pipe or C beam experiences peak relative displacement. Relative displacement, defined as the difference in displacement between a point and its support, serves as an indicator of deformation. It is evident that the pipe exhibits significantly greater deformation compared to other components, attributed to its connection solely at the bottom to the support hanger. Conversely, the bottom C-beam shows greater deformation than the top C-beam due to the connection of braces to the top C-beam, which restricts its translational freedom.

**Fig 23:**
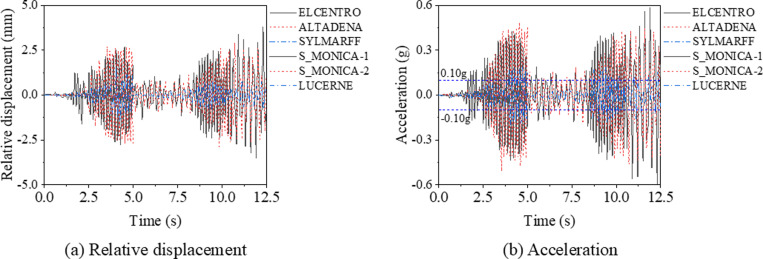
Relative displacement and acceleration of support hanger under different earthquake waves.

Fig 23 presents the time history curves of the relative displacement and acceleration at the center of the bottom C beam, with key values summarized in Table 3. As shown in Fig 23, the maximum deformation and acceleration of the structure occur after the earthquake acceleration has peaked. Notably, the maximum acceleration of the bottom C beam significantly exceeds the peak value of the input earthquake wave. As shown in Table 3, for the S_MONICA-1 wave scenario, the maximum acceleration and maximum relative displacement are recorded at 0.59g and 3.51 mm, respectively. Furthermore, the maximum von Mises stress reaches 188.1 MPa, which is below the yield stress of 235 MPa. These values are considerably higher than those obtained from other earthquake waves; the maximum von Mises stress, maximum acceleration, and maximum relative displacement are 3.3, 4.2, and 4.3 times those of the LUCERNE wave, respectively. It indicates that the shape of seismic wave (with the same maximum accelerate) has a significant effect on the seismic response of the bracing system. Consequently, the subsequent parametric study will utilize the S_MONICA-1 earthquake wave, due to the maximum responses of stress and displacement. However, to reduce computational expense, the data beyond the first 7.5 seconds is omitted, as the seismic response is considerably smaller than its peak values, as shown in Fig 23.

**Table 3:** Summary of the key results for support hanger under various types earthquake waves.

Earthquake wave	$\sigma_{mises}$ (MPa)	Center of the top C beam		Center of the bottom C beam	
		$A_{top}$ (g)	$D_{top}$ (mm)	$A_{bot}$ (g)	$D_{bot}$ (mm)
ELCENTRO	140.7	-0.08	0.019	-0.45	2.68
ALTADENA	139.0	-0.07	0.023	0.49	2.69
SYLMARFF	87.4	-0.07	0.019	0.25	-1.46
S_MONICA-1	188.1	-0.07	-0.035	0.59	-3.51
S_MONICA-2	148.4	0.09	-0.017	0.48	-2.89
LUCERNE	57.5	-0.12	0.008	-0.14	0.81

Note: $\sigma_{mises}$ is the maximum the von mises of the structure; $A_{top}$ and $D_{top}$ are the maximum acceleration and the relative horizontal displacement of the center of the top C beam; $A_{bot}$ and $D_{bot}$ are the maximum acceleration and the relative horizontol displacement of the center of the bottom C beam.

#### Pipeline.

**Fig 24:**
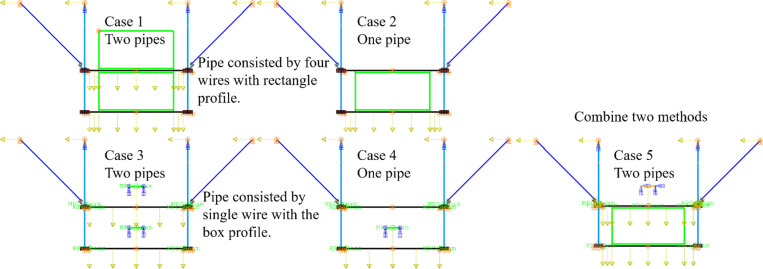
Pipe with different simplification methods.

Fig 24 presents numerical models incorporating pipes, constructed using two distinct simplification methods. The first method, as detailed in Section Brief of the numerical mode, is retained for comparison, while the second method simplifies the pipe to a single wire connected to the C beam via a multi-point constraint (MPC). It is evident that the bottom pipe, being connected only to the bottom beam, contributes minimally to the lateral stiffness of the support hanger and does not adequately represent the dynamic behavior of its four plates. Additionally, the models consider whether one or two pipes are included. To further elucidate the contribution of the top pipe, a model combining both simplification methods has been devised.

**Fig 25:**
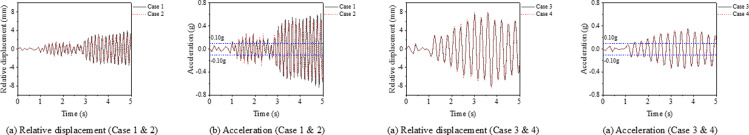
Relative displacement and acceleration of support hanger models.

Fig 25 illustrates the seismic response of structures from different models. It is evident that the presence of the pipe significantly influences the deformation and acceleration responses. The outcomes from Case 1, Case 2, and Case 5 (referred to as Group 1) are very similar, and they are markedly different from the data obtained in Case 3 and Case 4 (referred to as Group 2). Models in Group 1 display significantly less deformation response compared to those in Group 2, while the acceleration response shows an inverse trend. This suggests that the interaction between the pipe and the support hanger is crucial for accurately assessing its seismic response. Additionally, the maximum responses of von Mises stress, deformation, and acceleration for these models are summarized in Table 4. A comparison between Case 1 and Case 2 reveals that adding the top pipe (which doubles the design load of Case 2) results in an increase in the maximum von Mises stress, maximum relative displacement, and maximum acceleration by 24.7 MPa (+14.5%), 0.07g (+13.2%), and 0.44 mm (+14.0%), respectively. This is attributed to the top pipe being settled at the top C beam, which is supported by the braces, and its center of gravity being much closer to the top constraints. Therefore, it is more favorable to place a single pipe on the top C-beam, potentially eliminating the need for the bottom C-beam.

**Table 4:** Summary of the key results for support hanger models.

Earthquake wave	$\sigma_{mises}$(MPa)	Center of the top C beam		Center of the bottom C beam	
		$A_{top}$ (g)	$D_{top}$ (mm)	$A_{bot}$ (g)	$D_{bot}$ (mm)
Case 1	194.7	-0.07	-0.029	0.61	-3.59
Case 2	170.0	-0.07	-0.015	0.53	-3.15
Case 3	262.7	-0.09	0.016	-0.33	7.78
Case 4	262.6	0.10	0.016	-0.35	7.97

#### Peak acceleration.

**Fig 26:**
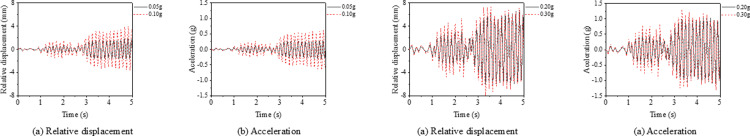
Relative displacement and acceleration of support hanger under different peak accelerations.

Fig 26 presents the time history curves of the relative displacement and acceleration at the center of the bottom C beam. It is evident that both the relative displacement and acceleration responses increase significantly with the peak acceleration of the input earthquake wave. As shown in Table 5, when the peak acceleration ranges from 0.05g to 0.30g, the maximum relative displacement and acceleration rise from 1.71 mm (0.29g) to 7.31 mm (1.30g), respectively. However, when the peak acceleration reaches or exceeds 0.20g, the maximum von Mises stress surpasses the yield stress of 235 MPa. This is attributed to the relatively low stiffness of the tie rod under the top C beam, where the maximum von Mises stress is observed. Consequently, for seismic supports designed to withstand large earthquakes, it is necessary to reinforce the substructure beneath the top C beam.

**Table 5:** Summary of the key results for support hanger under various peak accelerations.

$A_{\max}$(g)	$\sigma_{mises}$(MPa)	Center of the top C beam		Center of the bottom C beam	
		$A_{top}$ (g)	$D_{top}$ (mm)	$A_{bot}$ (g)	$D_{bot}$ (mm)
0.05	105.4	-0.04	-0.015	0.29	-1.71
0.10	194.7	-0.07	-0.029	0.61	-3.59
0.20	257.7	-0.14	-0.055	1.07	-6.43
0.30	262.7	-0.25	-0.102	-1.30	7.31

#### Connector thickness.

**Fig 27:**
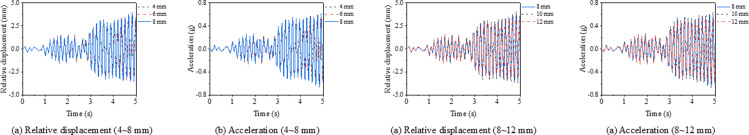
Relative displacement and acceleration of support hanger under different connector thicknesses.

Fig 27 shows the time history curves of the relative displacement and acceleration at the center of the bottom C beam, with the corresponding maximum von Mises stress, relative displacement, and acceleration values detailed in Table 6. Observations indicate that the thickness of the connector has a minimal impact on the seismic response of the support hanger. The coefficients of variation (CV) for the maximum von Mises stress, relative displacement, and acceleration are 0.067, 0.041, and 0.039, respectively. Consequently, for small to moderate earthquakes, the effect of connector thickness on the seismic response of support hanger can be considered negligible.

**Table 6:** Summary of the key results for support hanger under various connector thicknesses.

Thickness (mm)	$\begin{array}{c} \sigma_{mises} \end{array}$(MPa)	Center of the top C beam		Center of the bottom C beam	
		$\begin{array}{c} A_{top} \end{array}$ (g)	$\begin{array}{c} D_{top} \end{array}$ (mm)	$\begin{array}{c} A_{bot} \end{array}$ (g)	$\begin{array}{c} D_{bot} \end{array}$ (mm)
4	174.4	-0.07	-0.033	0.57	-3.39
6	194.5	-0.07	-0.030	0.58	-3.46
8	194.7	-0.07	-0.029	0.61	-3.59
10	202.5	-0.07	-0.030	0.63	-3.73
12	170.6	-0.07	-0.028	0.57	-3.37
Ave	187.3	–	–	0.59	-3.51
Std	12.5	–	–	0.024	0.135
CV	0.067	–	–	0.041	0.039

Ave means the average value; Std represents the standard deviation; CV is the coefficient of variation.

#### Brace angle.

**Fig 28:**
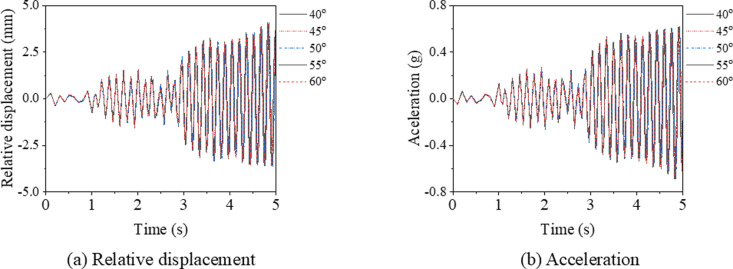
Relative displacement and acceleration of support hanger under different brace angles.

Fig 28 and Table 7 demonstrate that there is a negligible difference in the seismic responses among models with brace angles ranging from 40° to 60°. The coefficients of variation (CV) for the maximum von Mises stress, relative displacement, and acceleration are 0.032, 0.010, and 0.008, respectively. Consequently, for small or moderate earthquakes, the influence of the brace angle on the seismic response of support hanger can be considered minimal.

**Table 7:** Summary of the key results for support hanger under various brace angles.

Thickness (mm)	$\sigma_{mises}$(MPa)	Center of the top C beam		Center of the bottom C beam	
		$A_{top}$ (g)	$D_{top}$ (mm)	$A_{bot}$ (g)	$D_{bot}$ (mm)
40^∘^	199.6	-0.07	-0.032	0.62	-3.64
45^∘^	194.7	-0.07	-0.029	0.61	-3.59
50^∘^	184.9	-0.07	-0.037	0.61	-3.63
55^∘^	202.3	-0.07	-0.041	0.61	-3.60
60^∘^	201.1	-0.07	-0.064	0.60	-3.56
Ave	196.5	–	–	0.61	-3.60
Std	6.4	–	–	0.006	0.029
CV	0.032	–	–	0.010	0.008

#### Spacing of support hanger.

**Fig 29:**
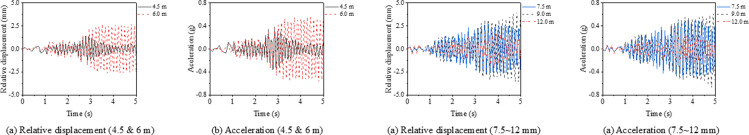
Relative displacement and acceleration of support hanger under different spaces.

**Fig 30:**
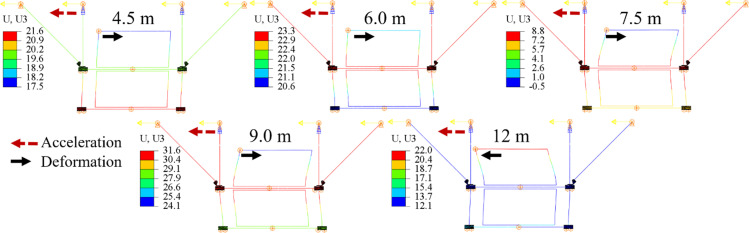
Displacement contours of models with different spacings (Deformation scale factor: 10).

Figs 29 and 30 illustrate the relative displacement and acceleration responses of the support hanger when subjected to different spacings. It is evident that the seismic response of the structure increases significantly with the increase in spacing. As shown in Table 8, when the spacing expands from 4.5 m to 9.0 m, the maximum von Mises stress, relative displacement, and acceleration rise from 77.9 MPa, 0.36g, and 1.46 mm to 194.7 MPa, 0.61g, and 3.59 mm, respectively. However, with the spacing increased to 12.0 m, the maximum von Mises stress, relative displacement, and acceleration are decreased to 103.6 MPa, 0.24g, and 1.64 mm, respectively. For that case, the acceleration and deformation of the upper pipe (the left top corner) shows same direction, which lead to a significant smaller relative displacement of the structure, as shown in Fig 30. Therefore, the stress and acceleration are also reduced. This indicates that the seismic response of support hanger is highly sensitive to the spacing between bracings.

**Table 8:** Summary of the key results for support hanger under various design loads.

Spacing (mm)	$\sigma_{mises}$ (MPa)	Center of the top C beam		Center of the bottom C beam	
		$A_{top}$ (g)	$D_{top}$ (mm)	$A_{bot}$ (g)	$D_{bot}$ (mm)
4.5	77.9	-0.07	-0.013	-0.36	1.46
6.0	133.0	-0.08	-0.023	0.55	-2.62
7.5	141.5	-0.07	-0.026	0.49	-2.67
9.0	194.7	-0.07	-0.029	0.61	-3.59
12.0	103.6	-0.07	-0.035	-0.24	1.64

## Conclusion

In this study, the authors have developed a novel finite element model to assess the pushover and seismic performance of support hanger systems. Drawing from the numerical simulation results and discussions presented in this paper, the following conclusions can be summarized: the parameters of connector thickness, connector angle, brace angle, and the spacing of support hanger differentially affect the pushover performance and seismic response of the support hanger. Connector thickness, connector angle, and brace angle significantly influence the pushover performance but have a limited impact on the seismic response. Conversely, the spacing of support hanger has a more pronounced effect on the seismic response. Additionally, peak acceleration significantly influences the seismic response. Therefore, selecting an appropriate earthquake scenario and peak acceleration is crucial for accurately evaluating the seismic performance of support hanger systems.
